# Pure Milk

**Published:** 1901-07-06

**Authors:** 


### PURE MILK.

In two directions attempts are about to be made to improve the condition of the milk supplied to the public. One applies to the whole kingdom, the other is a mere local movement initiated by the Borough Council of Camberwell, but on the whole the latter is the more interesting and important. Mr. Hanbury, President of the Board of Agriculture, recently announced in Parliament that after carefully considering the evidence submitted to the departmental committee, and their reports, he proposed to fix a standard under which for purposes of the Sale of Food and Drugs Act a presumption would be raised that milk was not genuine unless it contained 3 per cent. of milk fat and 8·5 per cent. of non-fatty milk solids. He stated that regulations were being prepared, and that he proposed that they should come into force on August 1st. This is a fair standard. It may now and again act hardly upon the small farmer with a single cow, but the ordinary dealer who mixes the milk of many cows ought to be able to furnish milk often even better than the standard suggested. We are afraid, however, that it will be difficult to prevent the dealer from diluting his milk with "skim," which he will now do without fear, so long as he keeps just above the standard. A much more important innovation is that taken in Camberwell, where it has been decided to inform the milk dealers that after the lapse of six months from the notice now to be given them, the Borough Council will take samples of milk for examination and will prosecute if these samples are found to be contaminated with tubercle bacilli. As a preliminary warning it has been arranged to have a considerable number of samples examined, and for the results to be made known to the vendors, so that they shall have an opportunity of seeking a more healthy source of supply in case their milk is found to be so contaminated. This is quite a new departure, and is a somewhat curious outcome of recent decisions in regard to the sale of arsenicated beer—an article not "of the nature, quality, and substance" asked for by the purchasers. The suggestion is that if a minute contamination of beer with arsenic, of the presence of which neither seller nor purchaser was aware, can render the seller liable, so can an equally minute contamination with tubercle bacilli. If the Camberwell authorities turn out to be right, their action will make for purity in milk. We fancy, however, that the first to profit by the innovation will be the lawyers and the noble army of experts.

